# Prenatal or Childhood Serum Levels of Vitamin D and Dental Caries in Paediatric Patients: A Systematic Review

**DOI:** 10.3290/j.ohpd.a45089

**Published:** 2020-09-04

**Authors:** Cátia Carvalho Silva, Rita Mendes, Maria da Conceição Manso, Sandra Gavinha, Paulo Melo

**Affiliations:** a PhD Student at Faculty of Dentistry, University of Porto, Porto, Portugal; Invited Assistant Professor, Faculty of Health Sciences, University Fernando Pessoa, Porto, Portugal. Study concept and design, acquisition, analysis and interpretation of data, drafted the article.; b Invited Assistant Professor, Faculty of Health Sciences, University Fernando Pessoa, Porto, Portugal. Data acquisition, drafted the article.; c Associate Professor, Faculty of Health Sciences, University Fernando Pessoa University Fernando Pessoa Energy, Environment and Health Research Unit (FP-ENAS), Porto, Portugal, and Associated Laboratory for Green Chemistry (LAQV/REQUIMTE), University of Porto, Porto, Portugal. Study design, revised the article critically for important intellectual content, final approval of the version to be submitted.; d Associate Professor, Faculty of Health Sciences, University Fernando Pessoa, Porto, Portugal. Data analysis and interpretation, revised the article critically for important intellectual content, final approval of the version to be submitted.; e Associate Professor, Faculty of Dentistry, University of Porto, Porto, Portugal; Chair, Department of Oral Public Health, Institute of Public Health, University of Porto; Epidemiology Research Unit (EPIUnit), Porto, Portugal. Study concept and design, data analysis and interpretation, revised the article critically for important intellectual content, final approval of the version to be submitted.

**Keywords:** dental caries, vitamin D, 25-hydroxyvitamin D, children, preventive dentistry

## Abstract

**Purpose:**

To assess the association between low prenatal or childhood levels of 25-hydroxyvitamin D (25(OH) D) and dental caries experience in children.

**Materials and Methods:**

PubMed, B-On, Web of Science, Scopus, and Cochrane Library databases were searched. The inclusion criteria were randomised controlled trials, cohort and cross-sectional studies published between 1998 and 2019; caries outcomes expressed as prevalence or based on the decayed missing and filled index for primary and permanent teeth/surfaces; and vitamin D levels assessed by laboratory analysis. Two authors independently selected studies, collected data, and assessed risk of bias. The quality of the studies was also assessed. A narrative synthesis of the studies was performed without quantitative pooling of data due to clinical and methodological heterogeneity.

**Results:**

Out of 399 studies identified, 13 were included in the data synthesis. Even though many of the included studies had a cross-sectional design, 11 were considered high quality. The studies indicated that vitamin D has an important role in caries experience, but also revealed that vitamin D levels equal to or above 75 nmol/l seem to be more closely related to caries experience than the reference value of the Institute of Medicine.

**Conclusion:**

Evidence of an association exists between low 25(OH) D levels (<75 nmol/l) and caries experience in children. Hence, low vitamin D levels should be considered a potential factor associated with caries in children. Clinicians should be aware that good prenatal nutrition and early childhood diet might influence caries experience.

Supplementary information

**Table S1:** Search strategy

MEDLINE/Pubmed – 54 articles	
("vitamin d"[MeSH Terms] OR "vitamin d"[All Fields] OR "ergocalciferols"[MeSH Terms] OR "ergocalciferols"[All Fields]) AND ("dental caries"[MeSH Terms] OR ("dental"[All Fields] AND "caries"[All Fields]) OR "dental caries"[All Fields]) AND ("child"[MeSH Terms] OR "child"[All Fields] OR "children"[All Fields]) AND ("1998/01/01"[PDAT]: "2019/10/30"[PDAT])	
Web of Science – 51 articles	
(TS=(*Vitamin D AND dental caries AND child)) AND LANGUAGE: (English) AND TYPE OF DOCUMENTS: (Article). Indexes=SCI-EXPANDED, SSCI, A&HCI, CPCI-SSH, ESCI, CCR-EXPANDED, IC. Time Period=1998-2019.	
B-ON – 216 articles	
(Academic Search Complete (198); Gale in Context: Science (11); Directory of Open Access Journals (3) and Science Direct (4) Research method: Boolean/Phrase (“dental caries AND Vitamin d AND Children) **Filters:** Publication Year 1998.01.01-2019.10.31. Language: English. Subject: Dentistry; Health and Medicine; Public Health. **Bibliographic expanders**: Apply related words; Apply equivalent subjects; Search also in the full text of the articles. Type of data sources: Academic journals. **Restrict by SubjectEDS:** oral health; infant nutrition; dentistry; caries; pediatrics; dietary supplements; public health; children’s health; vitamin D deficiency; children, vitamin D; dental caries; epidemiology; dental caries in children.	
Scopus – 67 articles	
TITLE-ABS-KEY ( ( dental AND caries OR caries ) AND ( vitamin AND d OR 25-hydroxyvitamin AND d ) AND child ) AND DOCTYPE ( ar ) AND PUBYEAR > 1997	
Cochrane Central Register of Controlled Trials – 9 articles	
"caries" in Title Abstract Keyword AND vitamin D in Title Abstract Keyword AND "Child" in Title Abstract Keyword - with Publication Year from 1998 to 2019, with Cochrane Library publication date Between Jan 1998 and Oct 2019, in Trials (Word variations have been searched)

**Table S2:** List of articles selected for full text analysis and reasons for exclusion

Article title	Reasons for exclusion
1. Analysis of the association between polymorphisms in the vitamin D receptor (VDR) gene and dental caries in a Chinese populations Hu XP, Li QZ, Zhou JY, Yu ZH, Zhang JM, Guo ML. Analysis of the association between polymorphisms in the vitamin D receptor (VDR) gene and dental caries in a Chinese population. Genet Mol Res 2015; 14(3):11631-8.	This study investigated only the relationship between polymorphisms in the vitamin D receptor gene and caries; laboratory assessment of 25(OH) D levels was not performed; participants over 12 years of age.
2. Assessment of Caries Experience, Enamel Defects, Feeding Types and Area of Residency in Children with Nutritional Rickets.s Jameel S and Al-Rawi N. Assessment of Caries Experience, Enamel Defects, Feeding Types and Area of Residency in Children with Nutritional Rickets. International Journal of Medical Research & Health Sciences 2018; 7(9): 59-65.	In this study, authors assessed the outcome in children with vitamin D nutritional deficiency rickets (children with systemic condition: exclusion factor); laboratory assessment of 25(OH) D levels was not performed.
3. Association between Single Nucleotide Polymorphisms in Vitamin D Receptor Gene Polymorphisms and Permanent Tooth Caries Susceptibility to Permanent Tooth Caries in Chinese Adolescents Yu M, Jiang QZ, Sun ZY, Kong YY, Chen Z. Association between Single Nucleotide Polymorphisms in Vitamin D Receptor Gene Polymorphisms and Permanent Tooth Caries Susceptibility to Permanent Tooth Caries in Chinese Adolescent. Biomed Res Int 2017: 4096316.	This study investigated only the relationship between polymorphisms in the vitamin D receptor gene and dental caries susceptibility; laboratory assessment of 25(OH) D levels was not performed.
4. Association of Alimentary and Nutritional Status with Caries in Children of Leon, Mexico Guizar JM, Muñoz N, Amador N, Garcia G. Association of Alimentary and Nutritional Status with Caries in Children of Leon, Mexico. Oral Health Prev Dent 2016; 14:563-569.	Laboratory assessment of 25(OH) D levels was not performed.
5. Child nutrition and oral health in Ulaanbaatar. Nutrition Research Karvonen HM, Nuutinen O, Uusitalo U, Sorvari R, Ihanainen M. Child nutrition and oral health in Ulaanbaatar. Nutrition Research 2003; 23:1165-1176.	Laboratory assessment of 25(OH) D levels was not performed.
6. Combined deficiencies of 25-hydroxyvitamin D and anemia in preschool children with severe early childhood caries: A case control study Deane S, Schroth RJ, Sharma A, Rodd C. Combined deficiencies of 25-hydroxyvitamin D and anemia in preschool children with severe early childhood caries: A case control study. Pediatrics & Child Health 2018; 23(3):e40-e45.	This article is a re-analysis of a previously described cross-sectional study [Schroth et al, 2013 (included in this systematic review)] using the original data set.
7. Dietary phosphorus burden increases cariogenesis independent of vitamin D uptake Goodson JM, Shi Ping, Humena CH, Haq A, Razzaque MS. Dietary phosphorus burden increases cariogenesis independent of vitamin D uptake. J Steroid Biochem Mol Biol 2017; 167:33-38.	Laboratory assessment of 25(OH) D levels was not performed.
8. Early Childhood caries with the Perspective of Pediatrician Bucak IH, Çalisir M, Almis H, Ozturk AB, Turgut M. Early Childhood caries with the Perspective of Pediatrician. J Clin Anal Med 2016; 7(5):614-617.	Laboratory assessment of 25(OH) D levels was not performed.
9. Estimation of vitamin d levels in children with and without early childhood caries – A case control study Jayakumar A, Gurunathan D, Subramainan EMG. Estimation of vitamin D levels in children with and without early childhood caries – A case control study. Indian Journal of Public Health Research & Development 2018; 9(11):51-56.	ASA II patients are included in this study (children with systemic condition: exclusion factor).
10. Evaluation of an interdisciplinary preventive programme for early childhood caries: findings of a regional German birth cohort study Wagner Y, Heinrich-Weltzien R. Evaluation of an interdisciplinary preventive programme for early childhood caries: findings of a regional German birth cohort study. Clin Oral Invest 2016; 20(8):1943-1952.	Laboratory assessment of 25(OH) D levels was not performed.
11. Fluoride/vitamin D tablet supplementation in infants-effects on dental health after 10 years Kühnisch J, Thiering E, Heinrich-Weltzien R, Hellwing E, Hickel R, Heinrich J. Fluoride/vitamin D tablet supplementation in infants-effects on dental health after 10 years. Clin Oral Invest 2017; 21(7):2283-2290.	Laboratory assessment of 25(OH) D levels was not performed.
12. Foetal, neonatal and child vitamin D status and enamel hypomineralization van der Tas JT, Elfrink MEC, Heijboer AC, Rivadeneira F, Jaddoe VWV, Tiemeir H, Schoufour JD, Moll HA, Ongkosuwito EM, Wolvius EB, Voortman T. Foetal, neonatal and child vitamin D status and enamel hypomineralization. Community Dent Oral Epidemiol 2018; 46(4):343-351.	The outcomes evaluated were molar incisor hypomineralization and hypomineralised second primary molars; dental caries was not evaluated.
13. Higher vitamin D intake during pregnancy is associated with reduced risk of dental caries in young Japanese children Tanaka K, Hitsumoto S, Miyake Y, Okubo H, Sasaki S, Miyatake N, Arakawa M. Higher vitamin D intake during pregnancy is associated with reduced risk of dental caries in young Japanese children. Ann Epidemiol 2015; 25(8):620-625.	The authors considered only the daily intake of vitamin D, laboratory assessment of 25(OH) D levels was not performed; there was no information about who evaluated the outcome: “…at public health center, the presence of dental caries was assessed by visual examination without the use of radiographs.”
14. Prenatal vitamin D and enamel hypoplasia in human primary maxillary central incisors: A pilot study Reed SG, Voronca D, Wingate JS, Murali M, Lawson AB, Hulsey TC, Ebeling MD, Hollis BW, Wagner CL. Prenatal vitamin D and enamel hypoplasia in human primary maxillary central incisors: A pilot study. Pediatr Dent J 2017; 27(1):21-28.	The outcome evaluated was only enamel hypoplasia.
15. Prevalence and risk factors for parental-reported oral health of Inuit preschoolers: Nunavut Inuit Child Health Survey, 2007-2008 Pacey A, Nancarrow T, Egeland GM. Prevalence and risk factors for parental-reported oral health of Inuit preschoolers: Nunavut Inuit Child Health Survey, 2007-2008. The International Journal of Rural and Remote Health Research, Education, Practice and Policy 2010; 1368:1-12.	This study did not directly associate 25(OH) D levels with dental caries; outcome was evaluated by questionnaires; laboratory assessment of 25(OH) D levels was not performed.
16. Prevalence of Caries among Preschool-Aged Children in a Northern Manitoba Community Schroth RJ, Smith PJ, Whalen JC, Lekic C, Moffatt MEK. Prevalence of Caries among Preschool-Aged Children in a Northern Manitoba Community. Journal of the Canadian Dental Association 2005; 71(1):27-27f.	Laboratory assessment of 25(OH) D levels was not performed.
17. Re-Examining the Association between Vitamin D and Childhood Caries Dudding T, Thomas SJ, Duncan K, Lawlor DA, Timpson J. Re-Examining the Association between Vitamin D and Childhood Caries. PLOS ONE 2015; 10(12):e0143769. doi: 10.1371/jornal.pone.0143769.eCollection 2015.	The outcome was measured based on questionnaires.
18. Serum cotinine, vitamin D exposure levels and dental caries experience in U.S. adolescents Akinkugbe AA, Moreno O, Brickhouse TH. Serum cotinine, vitamin D exposure levels and dental caries experience in U.S. adolescents. Community Dent Oral Epidemiol 2019; 47(2):185-192.	Participants over 12 years of age.
19. The impact of dietary and lifestyle factors on the risk of dental caries among young children in Qatar Bener A, Al Darwish MS, Tewfik I, Hoffmann GF. The impact of dietary and lifestyle factors on the risk of dental caries among young children in Qatar. The Journal of the Egyptian Public Health Association 2013; 88:67-73.	Participants included in this study were between 6 and 15 years of age.
20. The relationship between vitamin D receptor gene polymorphisms and deciduous tooth decay in Chinese children Kong YY, Zheng JM, Zhang WJ, Jiang QZ, Yang XC, Yu M, Zeng SJ. The relationship between vitamin D recpetor gene polymorphisms and deciduous tooth decay in Chinese children. BMC Oral Health 2017; 17(1):111.	In this study it was investigated only the relationship between polymorphisms in the vitamin D receptor gene and dental caries experience; laboratory assessment of 25(OH) D levels was not performed.
21. The Role of Vitamin D Receptor Polymorphisms on Dental Caries Cogulu D, Onay H, Ozdemir Y, Aslan GI, Ozkinay F, Eronat C. The Role of Vitamin D Receptor Polymorphisms on Dental Caries. J Clin Pediatr Dent 2016; 40(3):211-4	In this study it was investigated the role of vitamin D receptor polymorphisms on dental caries; laboratory assessment of 25(OH) D levels was not performed.
22. Toward Preventing Enamel Hypoplasia: Modeling Maternal and Neonatal Biomarkers of Human Calcium Homeostasis Reed SG, Miller CS, Wagner CL, Hollis BW, Lawson AB. Toward Preventing Enamel Hypoplasia: Modeling Maternal and Neonatal Biomarkers of Human Calcium Homeostasis. Caries Res 2019; 30:1-13.	The only outcome evaluated was enamel hypoplasia; dental caries was not evaluated.
23. Vitamin D Receptor Taql Gene Polymorphisms and Dental Caries in Czech Children Izakovicova Holla L, Borilova Linhartova P, Kastovsky J, Bartosova M, Musilova K, Kukla L, Kukletova M. Vitamin D Receptor Taql Genr Polymorphisms and Dental Caries in Czech Children. Caries Res 2017; 51(1):7-11.	In this study it was investigated the relationship between polymorphisms in the vitamin D receptor gene and dental caries experience; participants over 12 years of age; laboratory assessment of 25(OH) D levels was not performed.
24. Impact of vitamin D on development on early childhood caries Ali N, Rahim A, Ali S, Iqbal MH. Impact of vitamin D on development on early childhood caries. Pak Armed Forces Med J 2017; 67(3):429-433.	Inconsistencies were found in the methodology; this study has no information about who evaluated the outcome.

Vitamin D is known mainly for its role in calcium homeostasis, but it has been associated with several conditions and diseases.^16,39^ The two main sources of vitamin D are endogenous synthesis of vitamin D3 following skin exposure to ultraviolet B radiation from sunlight and exogenous uptake from diet and supplementation. Despite seasonal variations, the endogenous metabolism is estimated to synthesize up to 90% of the body’s vitamin D.^25^ The serum concentration of 25-hydroxyvitamin D (25(OH) D) is an established biomarker of vitamin D from both sources.^17^

Vitamin D plays a critical role in oral health,^6^ as its deficiency is associated with significant changes in dental-oral-craniofacial structures, and possibly several oral health conditions.^6,9,43^ Episodes of malnutrition and vitamin D deficiency during primary and permanent tooth formation can result in an increased risk of caries.^1,4,19,43^

Dental caries is a dynamic process that occurs when demineralisation of dental hard tissues, triggered by a sugar-driven dysbiosis of the dental plaque microbiome, overwhelms remineralisation through protective factors in the mouth.^34^ Despite multiple studies on the association of low levels of vitamin D with a higher risk of caries, the exact underlying mechanisms have not yet been clarified.^2^ Nevertheless, since ameloblasts and odontoblasts are target cells for the active form of vitamin D (1,25-dihydroxyvitamin D), vitamin D deficiency during odontogenesis may result in developmental defects, such as enamel hypoplasia^18,28^ or hypomineralisation,^40^ leaving teeth vulnerable to caries.^6^ Furthermore, the literature supports the role of vitamin D in immune responses to pathogens,^4,10^ which may reduce caries risk by promoting the production of antimicrobial peptides such as cathelicidins and defensins.^27^ Alterations in saliva flow and composition have also been suggested.^35^ Moreover, vitamin D supplementation and ultraviolet-B radiation appear to play a role in caries experience.^6,8^

During pregnancy, prenatal vitamin D levels may influence the primary dentition and the development of early childhood caries, as suggested in a prospective study based on self-reported maternal dietary vitamin D intake.^38^ Adequate vitamin D levels early in life may also play a role in caries prevention.^19^ Recent studies have investigated the correlation between caries and 25(OH) D levels, which helps determine vitamin D status.^17^ A pilot study reported a direct correlation of low prenatal levels of vitamin D with enamel hypoplasia in primary maxillary central incisors.^28^ On the other hand, a Mendelian randomised study conducted in a large sample of European children did not observe any correlation between 25(OH) D and caries.^4^ Nevertheless, a review of systematic reviews and meta-analyses that included observational studies and randomised trials addressing vitamin D and its health benefits suggested that vitamin D supplementation could help decrease caries experience.^39^

Despite the great variety of published data, the results of epidemiological and intervention studies investigating the association between vitamin D and caries remain inconclusive.^4,39^ Moreover, there is only one systematic review on this subject,^19^ which included controlled clinical trials addressing the role of supplemental dietary vitamin D or ultraviolet radiation in caries prevention when compared with no supplementation. It did not impose restrictions on the method of treatment or participant characteristics. That study showed that vitamin D could be a promising agent in caries prevention. Namely, those authors concluded with low certainty that vitamin D supplementation in childhood could reduce caries experience.^19^

In this context, the present systematic review aims to assess the association between higher caries experience and low prenatal or childhood serum levels of vitamin D by comparing children with these conditions with those having adequate 25(OH) D concentrations. Therefore, we aimed to answer the question: Are insufficient or inadequate prenatal/childhood vitamin D levels associated with higher caries experience compared with adequate/sufficient levels of vitamin D?

## Materials and Methods

### Study Design

The clinical question was formulated based on the PECO (Population, Exposure, Comparison, Outcome) strategy (Table 1). A detailed study protocol was established to determine the objectives, hypotheses to be tested, interest groups, and the proposed methods and criteria to be used to identify and select relevant studies as well as analyse information.

**Table 1:** PECO strategy used to evaluate the scientific evidence on dental caries experience in children who had low prenatal/childhood vitamin D levels

Parameter	Evaluation
Participants (P)	Children under the age of 13 years without any systemic disease
Exposure (E)	Insufficient† or inadequate‡ prenatal/childhood serum levels of vitamin D, (25(OH) D <75 nmol/l or 25(OH) D <50 nmol/l, respectively) measured by laboratory methods
Comparison (C)	Adequate/sufficient prenatal/childhood serum levels of vitamin D (25(OH) D ≥50 nmol/l or 25(OH) D ≥75 nmol/l) measured by laboratory methods
Outcome (O)	Association between higher caries experience and insufficient or inadequate prenatal/childhood serum levels of vitamin D Outcome Measures: Caries experience in primary, mixed, and permanent dentition, measured as prevalence, dmft/dmfs or DMFT/DMFS (or individual components of one of these indexes), assessed clinically by health professionals (with or without radiographic diagnosis) Exclusion criteria: studies that reported outcomes based on questionnaires applied to caregivers, associated 25(OH) D levels with other dental outcomes but not with caries, or considered vitamin D intake without laboratory analysis of 25(OH) D serum levels

†Insufficient levels of vitamin D according to the Endocrine Society (25(OH) D <75 nmol/l);^15^ ‡Inadequate levels of vitamin D according to Institute of Medicine (25(OH) D <50 nmol/l).^29^ 25(OH) D: 25hydroxyvitamin D; dmft/dmfs: decayed, missing, and filled primary teeth/surfaces; DMFT/DMFS: decayed, missing, and filled permanent teeth/surfaces.

### Search Strategy and Data Sources

A systematic literature search was conducted by two independent reviewers (C.C.S. and P.M.) in the following electronic databases: MEDLINE (PubMed), B-ON (Academic Search Complete, Gale in Context: Science, Directory of Open Access Journals and Science Direct), Web of Science, Scopus, and Cochrane Central Register of Controlled Trials. The search strategy is presented in Table S1 (Supplementary Information). Blocks of search terms were designed and combined. The search was restricted to manuscripts written in English and published between January 1, 1998, and October 31, 2019. This time limitation was established because articles older than 1998 could lead to conclusions that are not representative of the current situation due to differences in methodology (ie, laboratory methods for assessing vitamin D levels), children’s lifestyle (ie, diets lower in carbohydrates and phosphate), access to oral healthcare, and exposure to fluoride.

The inclusion criteria were: (1) randomised controlled trials (RCTs), cohort studies, case-control studies or cross-sectional studies, (2) assessment of caries experience in children under 13 years old, (3) outcome clinically assessed by healthcare professionals in primary, mixed or permanent dentition, and (4) laboratory assessment of prenatal or childhood vitamin D serum levels. The exclusion criteria were: studies that assessed caries experience in children with systemic diseases; outcomes based on questionnaires applied to the caregivers; association of 25(OH) D levels with dental outcomes other than caries; and assessments based on vitamin D intake without laboratory analysis of 25(OH) D serum levels.

This systematic review interpreted vitamin D levels according to the 2011 Report of the Institute of Medicine (IOM)^29^ and to the Endocrine Society’s Clinical Practice Guideline.^15^ The IOM report considers that adequate levels of vitamin D range between 50 and 125 nmol/l, whereas the Endocrine Society states that the serum concentration of 25(OH) D should be equal or above 75 nmol/l.

### Resource Selection and Data Extraction

Two reviewers (C.C.S. and P.M.) conducted the initial screening of all retrieved titles and abstracts and read the full text of the eligible studies. In cases of disagreement, they consulted a third reviewer (S.G.), and the differences were resolved by consensus.

A previously designed chart (based on study design, population characteristics, levels of 25(OH) D, outcomes measures and results) was used by the two reviewers (C.C.S. and P.M.) to extract data, and another reviewer (M.C.M.) was consulted in cases of disagreement to reach a consensus. The following information was recorded: location of the study, design, method for assessing 25(OH) D levels, sample size, age of the participants, outcome and how it was measured, statistical analysis, adjustment for confounders, overall results, direction of the effect (if statistically significant), and strength of the association.

Data were extracted as described in the corresponding studies, including prevalence, decayed, missing and filled primary teeth/surfaces (dmft or dmfs) or decayed, missing and filled permanent teeth/surfaces (DMFT or DMFS indices, or individual components of either index separately), Pearson’s correlation coefficients (r) and odds ratios (OR) with respective confidence intervals (CI), p-values, or other summary effect measures.

### Risk of Bias

Two reviewers (C.C.S. and P.M.) evaluated the methodological quality of the studies using the Cochrane risk of bias tool for randomised controlled trials,^14^ the Newcastle-Ottawa scale for cohort studies,^41^ and a modified version of the latter tool for cross-sectional studies. The Newcastle-Ottawa scale comprises eight items grouped into three categories: selection (four items), comparability (one item), and outcome (three items). Studies are given a classification of up to nine points, with each item representing one point, except the comparability item, which may represent up to two points. The modified version of this scale used for cross-sectional studies has a similar number of categories, but the outcome category includes two items instead of three; thus, it scores up to eight points.^41^ Studies with a score above the median were classified as high-quality studies:^20^ cohort studies > 4.5 and cross-sectional studies > 4.

Heterogeneity among the included studies was evaluated based on parameters such as the conditions of caries assessment and the instruments used for it, laboratory methods for assessing 25(OH) D levels, and sample characteristics. A high degree of clinical and methodological heterogeneity was found between studies. The methods used for assessing 25(OH) D levels and caries were reported in different ways. The diverging cut-off points, the variety of statistical analyses, and the summary measures among studies, especially those concerning caries, precluded the performance of a meta-analysis. Thus, a narrative synthesis of the included studies was performed.

## Results

### Study Selection and Characteristics

**Fig 1:**
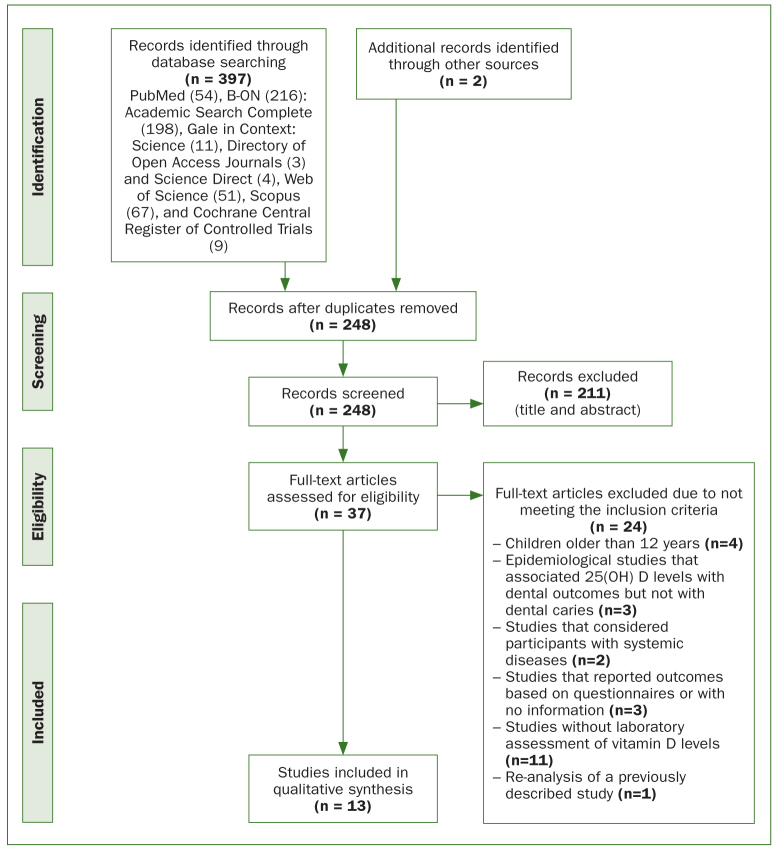
The Preferred Reporting Items for Systematic Reviews and Meta-Analyses^24^ (PRISMA) flow diagram.

The preferred reporting items for systematic reviews and meta-analyses (PRISMA)^24^ flow diagram in Fig 1 explains the methodological strategy followed for selecting the articles to include in this systematic review.

From the initial search of the electronic databases, 397 articles were selected, and two more were selected in the subsequent manual search of the selected references. After elimination of repeated articles, the reviewers read the abstracts of the resulting 248 articles and concluded that only 37 were relevant for this study, having selected them for integral text analysis. Then, 24 articles were excluded due to not fulfilling the inclusion criteria (Table S2, Supplementary Information). The final thirteen articles focused on the caries experience of children aged between 12 months and 12 years old and their serum levels of vitamin D.

The included studies are described in Table 2. This systematic review included only one randomised controlled trial,^26^ four cohort studies,^22,31,36,37^ and eight cross-sectional studies.^3,11,13,21,23,30,32,33^ Eight studies^3,22,26,30-32, 36,37^ evaluated the impact of 25(OH) D serum levels on caries experience in primary teeth, of which five^22,26,31,36,37^ assessed the impact of prenatal levels and three^3,30,32^ of childhood levels. Three studies^11,13,33^ assessed caries experience in mixed dentition and two studies in permanent dentition.^21,23^ Two studies^23,26^ evaluated caries experience in both primary and permanent teeth.

**Table 2:** Characteristics of included studies

Author/Year	Country	Study Design	Method for Assessing 25(OH) D levels	Group	N	Age	Dental Caries in Children							Statistics adjusted for con-founders	Caries Diagnosis Criteria	Outcome (OR; 95% CI; or p-value)
							Primary Dentition			Mixed Dentition Period		Permanent Dentition				
							Preva-lence (%)	dmft or dmfs mean±SD		Preva-lence (%)	dfs/DFS	Preva-lence (%)	DMFT or DMFS mean±SD			
Nørrisgaard et al^26^ (2019)^†^	Denmark	Double-blind, single-center, randomized clinical trial	Isotopedilution liquid-chromatography-tandem mass spectrometry	Supplementation during pregnancy Pregnant mother: 2800 IU/day Pregnant mother: 400 IU/day	496	6 years	20.1 20.2					4.7 3.8	Logistic regression^a^ – primary teeth: (participation in the long-chain ω-3 polyunsaturated fatty acids, sex, birth season, maternal serum vitamin D_3_ level at randomisation, and socioeconomic status) Logistic regression^b^ - permanent teeth: (model^a^ and number of erupted first permanent molars)		Cavitated Lesions (WHO, 2013)	No association found OR=1.01, 95%CI=0.65-1.59^a^ OR=1.32, 95%CI=0.41-4.48^b^ There was no association between high-dose vitamin D_3_ supplementation and caries in both dentitions OR=0.54, 95%CI=0.30-0.94^a^ OR=0.42, 95%CI=0.23-0.73^b^ The risk of enamel defects in the permanent and primary dentition was lower in the offspring of mothers who received high-dose vitamin D supplementation
Singleton et al^37^ (2019)^†^	Alaska (USA)	Cohort (retrospective)	Radioimmuno-assay			12-59 months		dmft scores					Multivariable linear regression^a^ (infant birth weight and gestational age), t-test^b^		Non-Cavitated and Cavitated Lesions (American Dental Association, 2015)	Association found Cord blood 25 (OH) D and caries in children between 12 and 35 months p=0.002^b^, 12-35mo children with cord blood 25(OH) D<30nmol/L had a mean dmft score twice as high as children with 25(OH) D ≥30nmol No association found Cord blood 25 (OH) D and caries in children between 36 and 59 months p=0.140^b^, there was no significant difference in dmft score of 36-59mo children with deficient vs. nondeficient cord blood 25(OH)D No significant differences in mean dmft scores for 12-35mo and 36-59mo children, whose mothers’ prenatal 25(OH) D levels were above or below 50nmol/L
				Umbilical cord 25(OH) D: <30nmol/L ≥30nmol/L	57			12-35mo 9.3 (1.1) 4.7 (0.9)	36-59mo 10.9 (1.0) 8.7 (1.1)							
				Pregnant Mother <50nmol/L ≥50nmol/L and <75nmol/L	76			9.0 (2.5) 7.4 (1.0)	14.4 (1.0) 10.1 (1.1)							
Silva et al^36^ (2019)^†^	Australia	Twin Cohort (prospective)	Chemical luminescence immunoassay	Pregnant mother at 28 weeks’ gestation Umbilical Cord The 25(OH) D concentrations as a continuous variable	329 241	6-7 years	Any Caries: 32.0 Advanced caries: 24.1						Linear regression ^a^, Multiple logistic regression^b^ (age, gender maternal obesity, maternal smoking in second or third trimester, chorionicity, birth vitamin D, water fluoridation, hypomineralised second primary molars); Multiple logistic regression^c^ (age, gender, maternal obesity, maternal vitamin D at 28 weeks, maternal smoking in second or third trimester, chorionicity, water fluoridation, hypomineralised second primary molars)		Any Caries: including non-cavitated lesions and/or past treatment Advanced Caries: established carious lesions with ICDAS codes 4-6 and/or past treatment	No association found Advanced Caries – Birth Vitamin D: OR=1.50, 95%CI =1.04-2.15, p=0.030^a^, as the vitamin D level at birth increases by 20nmol/L, the odds of dental caries increase 1.5-fold Advanced Caries Birth Vitamin D: OR=1.41, 95%CI =0.90-2.21, p=0.130^b^ Maternal Vitamin D: OR=1.49, 95%CI =0.90-2.45, p=0.120^c^ No association was found between maternal vitamin D or child vitamin D at birth and dental caries in primary canines and molars
Korun et al^22^ (2017)^†^	Cyprus	Cohort (prospective)	Electrochemica- luminescence immunoassay	Umbilical Cord: 25(OH) D: <75nmol/L* 28 ≥75nmol/L 6	50	12-24 months	ECC						t-test^a^, Logistic regression^b^ (child’s tooth brushing habits, mother’s tooth brushing habits and father’s dental status), Pearson correlation^c^		Non-Cavitated and Cavitated Lesions National Institute of Dental and Craniofacial Research, the Health Resources and Services Administration, and the Health Care Financing Administration - a report of a workshop (Drury et al, 1999)	Association found p=0.002^a^, Low cord blood level of 25(OH) D was significantly related to the development of ECC (compared with the control group: ECC=0) p=0.039, 95%CI: 0.832-0995^b^ r=-0.45, p=0.010^c^, dmft had a significantly negative correlation with the level of cord blood 25(OH) D
Schroth et al^31^ (2014)^†^	Canada	Cohort (prospective)	Radioimmuno-assay (DiaSorian®)	Pregnant mother 25(OH) D: <35nmol/L 1.6±2.3 (0-10) ≥35nmol/L 1.1±1.9 (0-9) <50nmol/L 1.0±1.9 (0-10) ≥50nmol/L 1.4±2.1 (0-9) <75nmol/L 1.4±2.2 (0-10) ≥75nmol/L 0.6±1.2 (0-4)	132	12 months		dt scores ECC					Aspin-Welch unequal-variance test^a^, logistic regression^b^ (serum metabolites, factors influencing vitamin D status (season), infant feeding practices, socioeconomic factors, dental status and dental behaviors), Backward logistic regression^c^ (income and employment status, infant feeding, season and oral hygiene practice), Poisson regression^d^ Logistics regression for ECC (excluding white spot lesions)		Non-Cavitated and Cavitated Lesions National Institute of Dental And Craniofacial Research, the Health Resources and Services Administration, and the Health Care Financing Administration - a report of a workshop (Drury et al, 1999)	Association found p=0.030^a^, Infants of mothers who had optimal 25(OH) D levels (≥75nmol/L) had a statistically lower dt score than those with mothers who had levels below this threshold OR=2.02, 95%CI=1.00-4.08, p=0.050^b^, low levels of 25(OH) D during pregnancy were significantly associated with ECC p=0.02^c^, lower 25(OH) D levels were significantly and independently associated with ECC r= -0.013, 95%CI=0.0085, p=0.002^d^, Lower 25(O) D levels were associated with higher dt scores
Chhonkar et al^3^ (2018)^‡^	India	Cross-sectional	–	Child 25(OH) D: <50nmol/L* >50nmol/L	60	3-6 years	S-ECC 69.05 5.56						t-test^a,^ simple linear regression^b^		Lack Of Information on S-ECC Definition	Association found p<0.0001^a^, the mean levels of serum 25(OH) D were compared between case (S-ECC>0) and control (S-ECC=0) groups and there was a statistically significant difference p<0.0001^b^, statistically significant inverse correlation between 25(OH) D levels and S-ECC
Schroth et al^32^ (2013)^‡^	Canada	Cross-sectional	Chemical- luminescence immunoassay	Child 25(OH) D: <35nmol/L 75.0 ≥50nmol/L 50.9 ≥75nmol/L 44.8	261	≤71 months	S-ECC						t-test^a^, logistic regression^b^ (age when started cleaning teeth, bottle feeding, general health, mean vitamin D, vitamin D drop usage, yearly income)		Definition for S-ECC (AAPD, 2010)	Association found p<0.001^a^, mean 25(OH)D levels were significantly lower among children with S-ECC than caries-free controls OR=1.01, 95%CI=1.00-1.02, p=0.040^b^, lower vitamin D mean levels were significantly associated with S-ECC
Schroth et al^30^ (2012)^‡^	Canada	Cross-sectional	-----	Child 25(OH) D: <25nmol/L 0.0 ≥25nmol/L 50.0 <75nmol/L 53.1 ≥75nmol/L 33.3	38	<72 months	S-ECC						t-test^a^, logistic regression (1st model: PTH, 25(OH)D)^b^ (2nd model: parental education and 25(OH)D)levels^c^		Definition for S-ECC (AAPD, 2010)	No association found in multivariate models p=0.032, (one tailed)^a^, children with S-ECC had significantly lower 25(OH) D levels than caries-free children OR=2.2, 95%CI=1.18-3.13, p=0.120^b^, 25(OH) D levels were not associated with S-ECC OR=1.7, 95%CI =0.90-2.5, p=0.190^c^, 25(OH) D levels were not associated with S-ECC
Gyll et al^11^ (2018)^§^	Sweden	Cross-sectional (prospec-tive)	Mass spectrometry	Child 25(OH) D: <50nmol/L 5.8 >50nmol/L 1.4	85	8 years					d_3___4_fs /D_3___4_FS		Chi-square^a^, logistic regression (1st model: number of teeth, tooth brushing, presence or absence of S. mutans, father’s educational level, region of residence)^b^ (2nd model: 1st model+ BMI, intake of a vitamin D supplement)^c^ (3rd model: 2nd model + skin type)^d^, Backward elimination^e^		Cavitated Lesions (WHO, 1997) D_3___4_FS/d_3___4_fs scores: 3-4 represent caries lesions into the dentin	Association found (not totally consistent) p=0.084^a^, vitamin D levels did not differ between children with or without caries OR=0.961, 95%CI=0.929-0.995, p=0.024^b^, higher vitamin D levels were significantly associated with less caries OR=0.962, 95%CI=0.928-0.998, p=0.037^c^, higher vitamin D levels were significantly associated with less caries OR=0.967, 95%CI=0.931-1.005, p=0.085^d^, attenuated the results slightly p=0.010^e^, 25(OH)D levels were independently associated with having caries
Schroth et al^33^ (2016)^§^	Canada	Cross-sectional	Chemical- luminescence immunoassay	Child 25(OH) D: <50nmol/L 68.50 ≥50nmol/L 54.80 <75nmol/L 56.40 ≥75nmol/L 43.60	1017	6-11 years							Multiple logistic regression (tooth brushing, visits to the dentist at least once a year, sugary drinks/day, milk/day, water fluoridation, household education, household income, dental insurance)^a,^ Backward elimination^b^		Lack of Information on Caries Definition	Association found OR=0.46, 95%CI=0.26-0.83, p=0.009^a^, levels of 25(OH) D≥50nmol/L were significantly and independently associated with lower adjusted odds for caries OR=0.68, 95%CI=0.40-1.14, p=0.130^a^, levels of 25(OH) D≥75nmol/L were not significantly and independently associated with lower adjusted odds for caries r= -0.91, 95%SE=0.28, p=0.008^b^, levels of 25(OH) D≥75nmol/L were significantly and independently associated with lower dmft/DMFT scores r= -0.80, 95%SE=0.42, p=0.060^b^, levels of 25(OH) D≥50nmol/L were not associated with lower caries scores
Herzog et ^al13^ (2016)^§^	USA	Cross-sectional	Radioimmuno-assay	Child 25(OH) D: <30nmol/L 2.37 30-49nmol/L 15.79 50-125nmol/L 81.46 >125nmol/L 0.38	1103	5-12 years							Multivariate logistic regression^a^(age, sex, race, ethnicity, ratio of family income to poverty threshold, sugar consumption)b		Cavitated Lesions	No association found OR=0.69, 95%CI=0.37-1.29), p=0.224^a^, no significant association between vitamin D levels and caries experience OR=0.65, 95%CI=0.27-1.52, p=0.296^b^, after adjustment for confounders, no significant association between vitamin D levels and caries experience
Kühnisch et al^23^ (2015)^¶^	Germany	Cross-sectional	Automated modular system	The 25(OH) D concentrations as a continuous predictor	1048	10 years		d_3_-_4_mfs: 41.6					D3-4MFS: 16.4	Poisson Hurdle Models: 25(OH) D levels were corrected for sampling date to normalize for seasonal variability. (Model 1^a^: sex, age and body mass index; Model 2^b^: (1) and socioeconomic factors; Model 3^c^: (2) and time spent in front of TV/PC in winter and summer)	Cavitated Lesions (WHO, 1997) D_3___4_/d_3___4_ scores: 3-4 represent caries lesions into the dentin	Association found (D3-4MFS) RR= 0.93 (0.88-0.99), p=0.032^a^ RR= 0.93 (0.87-0.99), p=0.023^b^ RR= 0.94 (0.89-0.99), p=0.019^c^ Higher 25(OH) D was associated with less dental caries in permanent teeth No association found (d3-4mfs) RR= 1.00 (0.98-1.01), p=0.661^a^ RR= 1.00 (0.98-1.01), p=0.718^b^ RR= 1.00 (0.98-1.01), p=0.718^c^ Higher 25(OH) D was not associated with less dental caries in deciduous teeth
Kim et al^21^ (2018)^¶^	Korea	Cross-sectional	Competitive protein binding	Child 25(OH) D: <50nmol/L 54.7 ≥50nmol/L 47.0	1688	10-12 years							Chi-square^a^, logistic regression (sex, household income, age, frequency of tooth brushing)^b^		Lack of Information on Caries Definition	No association found (dental caries experience) Association found (1st PM) p=0.0012^a^, children with levels of 25(OH) D<50nmol/L had higher caries experience than children with 25(OH) D≥50nmol/L p=0.006^a^, children with levels of 25(OH) D<50nmol/L had higher dental caries experience in permanent first molar than children with 25(OH) D≥50nmol/L OR=1.246, 95% CI=0.975-1.592, p=0.079^b^, there was no association between 25(OH) D levels and caries experience OR=1.295, 95% CI=1.020-1.644, p=0.034b, there was a significant association between 25(OH) D levels and first permanent molar caries experience

†Relationship between prenatal vitamin D and caries experience in children; ‡relationship between vitamin D in children and dental caries experience in primary dentition; §relationship between vitamin D in children and caries experience in mixed dentition; ¶Relationship between vitamin D in children and caries experience in permanent dentition. 25(OH) D: 25-hydroxyvitamin D; OR: odds ratio; CI: confidence interval; IU: international units; %: percentage; SD: standard deviation; dmft: decayed, missing and filled primary teeth; dmfs: decayed, missing and filled primary teeth surfaces; dt score: decayed primary teeth rate; dfs: decayed and filled primary teeth surfaces; DFS: decayed and filled permanent teeth surfaces; d3-4fs: decayed (codes 3 and 4 of the International Caries Detection and Assessment System (ICDAS)) and filled primary teeth surfaces; D3-4FS: decayed (codes 3 and 4 of the ICDAS) and filled permanent teeth surfaces; DMFT: decayed, missing and filled permanent teeth; DMFS: decayed, missing and filled permanent teeth surfaces; ECC: early childhood caries; S-ECC: severe early childhood caries; r: determination coefficient; mo: months old; 1st PM: permanent first molar; SE: standard error; RR: relative risk; *the values here shown result from a conversion from ng/ml (conventional units) to nmol/l (SI units) (1 ng/ml equals 2.5nmol/l); USA: no information available.

### Synthesised Findings

The RCT by Nørrisgaard et al^26^ revealed no relationship between caries in both dentitions and vitamin D3 supplementation in higher doses (2800 IU/day vs 400 IU/day [placebo group]) from the 24th pregnancy week to the 1st week post-partum. However, it found that a higher dose of vitamin D3 supplementation was associated with approximately 50% reduced odds of enamel defects in the offspring at 6 years of age.

Schroth et al^31^ verified that children with early childhood caries (ECC) had prenatal levels of 25(OH) D significantly lower than children with no cavitated lesions. Korun et al^22^ revealed a significant association between umbilical cord 25(OH) D levels <75 nmol/l and enamel hypoplasia, which in turn is associated with caries.^18,28^ They also reported that low umbilical cord 25(OH) D levels played a major role in the development of ECC and enamel hypoplasia.^22^ Tanaka et al^38^ had results similar to those two studies. On the other hand, Singleton et al^37^ verified that low levels of umbilical cord vitamin D (25(OH) D <30 nmol/l) were associated with a two-fold higher mean dmft score in children with 12–35 months of age but not in older ones (36-59 months). However, they did not observe an association between higher mean dmft scores in either of those age ranges and maternal levels of vitamin D under the cut-off value of 50 nmol/l. Similarly, Silva et al^36^ did not find an association between caries and a low maternal vitamin D level and a low child vitamin D level at birth in their adjusted analysis.

Three of the included studies^3,30,32^ analysed the association between vitamin D levels in children and their caries experience in primary teeth. In their pilot study, Schroth et al^30^ hypothesised and confirmed that children with severe early childhood caries (S-ECC) had lower levels of 25(OH) D than caries-free children. However, they found a significant association only at the bivariate level. In a later study,^32^ the same authors found that children with 25(OH) D <75 nmol/l were twice as likely to develop S-ECC. Although they verified the association for the IOM reference value,^29^ they did not verify it for values of 25(OH) D <35nmol/l. Chhonkar et al^3^ also compared the vitamin D levels of children with and without S-ECC and found a deficiency of 25(OH) D (<50 nmol/l) in 29 out of 30 children with S-ECC, compared with only 13 out of 30 in the control group.

Three studies^11,13,33^ focused on the association between 25(OH) D levels in children and their caries experience in mixed dentition. Schroth et al^33^ observed a representative sample of Canadian children with appropriate sampling and found an independent and significant association between levels of 25(OH) D ≥50 nmol/l (IOM reference cut-off value) and less adjusted odds of caries, but not for levels of 25(OH) D ≥75 nmol/l. However, when a backward elimination was undertaken, the final interaction revealed that both concentrations were significantly and independently associated with a lower risk of caries. Herzog et al^13^ also studied a representative national sample of children and did not find a statistically significant association between insufficient levels of 25(OH) D and caries experience (presence of at least one untreated decayed tooth or one restored tooth). More recently, Gyll et al^11^ studied the association between vitamin D concentration in 6-year-old children and their caries status two years later. Those authors found that vitamin D levels did not differ between children with and without caries at the bivariate level; at the multivariate level, the significant association depended on the confounder to which the model was adjusted. However, backward elimination revealed that lower levels of 25(OH) D were independently associated with having dentin caries lesions in primary and/or permanent dentition.

Regarding the association between vitamin D levels in children and their caries experience in permanent dentition, Kühnisch et al^23^ verified that higher vitamin D levels were associated with less caries in permanent but not in primary teeth; however, caries evaluation was not the main objective of that study. More recently, Kim et al^21^ found that the group with higher levels of vitamin D had a lower proportion of children with caries experience, particularly regarding the permanent first molars. Those authors found that, after adjusting the variables sex, age, household income, and toothbrushing frequency, children with 25(OH) D <50 nmol/l showed a 1.295 higher likelihood of having caries in the permanent first molars than children with levels of 25(OH) D ≥50 nmol/l.

### Risk of Bias

**Fig 2:**
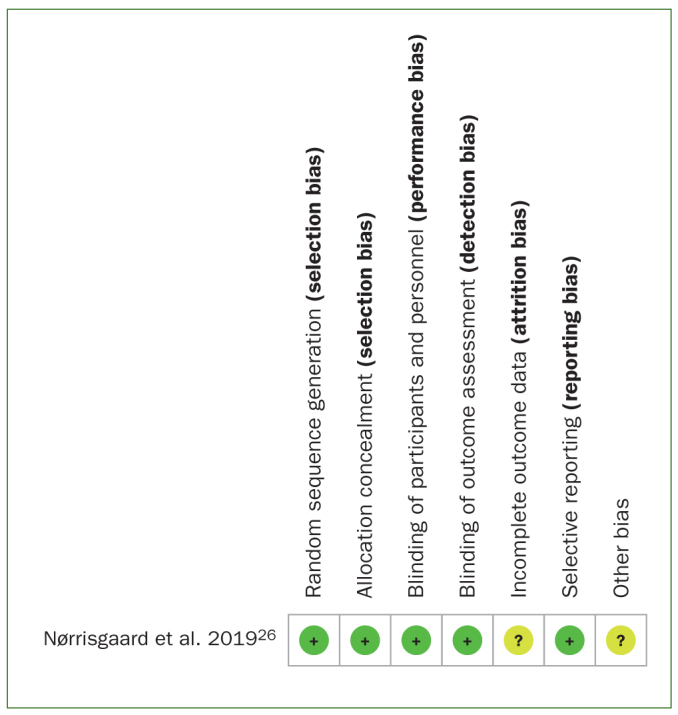
Risk of bias analysis of randomised controlled trials.^14^ (+): low risk of bias; (-): high risk of bias; (?): unclear risk of bias.

The risk of bias analysis (Fig 2) of the only RCT included^26^ demonstrated that the randomisation of participants was clear. The main shortcoming of that study was not describing the method used to conceal the allocation sequence in sufficient detail and whether the reported results included all outcomes. Nevertheless, the study was considered of high quality.

Regarding the assessment of the methodological quality of cross-sectional studies (Table 3), six out of eight studies were considered of high quality^11,13,21,23,32,33^ (score > 4). The most common drawbacks of cross-sectional studies were not reporting non-response rates^3,11,13,21,23,30,32,33^ and relying on non-representative^3,11,23,30,32^ and non-calculated samples.^3,11,23,30^ Another shortcoming was related to the external validity of the studies, as we considered a broad age range, varying from 12 months to 12 years old, and the laboratory method used to assess levels of vitamin D differed between studies. Also, only one study^3^ did not analyse the controlling confounders, and only two studies^3,30^ did not control the most important confounder (socioeconomic factor).

**Table 3:** Quality assessment of the cross-sectional studies included according to the modified Newcastle-Ottawa Scale^41^

Author, year	Selection				Comparability	Outcome		Score
	Representativeness of the sample^[1]^	Sample size^[2]^	Ascertainment of the exposure[3]	Non-respondents^[4]^	Adjustment of confusion^[5]^	Assessment of outcome^[6]^	Statistical test^[7]^	
Chhonkar et al, 2018^3^	(c)	(b)	(c)	(c)	-	*	*	2 (8)
Schroth et al, 2013^32^	(c)	*	*	(c)	**	*	*	6 (8)
Schroth et al, 2012^30^	(c)	(b)	(c)	(c)	*	*	*	3 (8)
Gyll et al, 2018^11^	(c)	(b)	*	(c)	**	*	*	5 (8)
Schroth et al, 2016^33^	*	*	*	(c)	**	*	*	7 (8)
Herzog et al, 2016^13^	*	*	*	(c)	**	*	*	7 (8)
Kühnisch et al, 2015^23^	(c)	(b)	*	(c)	**	*	*	5 (8)
Kim et al, 2018^21^	*	*	*	(c)	**	*	*	7 (8)

[1] (a) Truly representative of the average in the target population* (all subjects or random sampling); (b) somewhat representative of the average in the target population* (non-random sampling); (c) selected group of users; (d) no description of the sampling strategy. [2] (a) Justified and satisfactory*; (b) not justified. [3] (a) Validated measurement tool*; (b) non-validated measurement tool, but the tool is available or described *; (c) no description of the measurement tool. [4] (a) Comparability between the characteristics of respondents and non-respondents is established, and the response rate is satisfactory*; (b) the response rate is unsatisfactory, or the comparability between respondents and non-respondents is unsatisfactory; (c) no description of the response rate or the characteristics of responders and non-responders. [5] (a) The study controls for the most important factor (socioeconomic status)*; b) the study controls for any additional factor (season of measurement of 25(OH) D levels, ethnicity, age, household income, vitamin D supplement intake, regular milk drinker, body mass index)*. [6] (a) Independent blind assessment*; (b) record linkage*; (c) self-report; (d) no description. [7] (a)The statistical test used to analyze the data is clearly described and appropriate, and the measurement of the association is presented, including confidence intervals and the probability level (p-value)*; (b) the statistical test is not appropriate, not described or incomplete.

The cohort studies^22,31,36,37^ (Table 4) all failed by relying on a non-representative exposed cohort,^22,31,36,37^ and one cohort study^31^ had a follow-up period shorter than 12 months and a follow-up rate of less than 80%. Despite these flaws, the cohort studies had high quality (score > 4.5). The prospective nature of these studies^22,31,36^ strengthens their evidence, as it allows a blind evaluation of the relationship between concentrations of 25(OH) D during pregnancy and odontogenesis and caries experience in children. These cohort studies have the advantage of presenting a low risk of bias; however, the use of convenience samples may limit the generalisability of their results.

**Table 4:** Quality assessment of the cohort studies included according to the Newcastle-Ottawa Scale^41^

Author, year	Selection				Comparability	Outcome			Score
	Representativeness of the exposed cohort^[1]^	Selection of the non exposed cohort^[2]^	Ascertainment of exposure^[3]^	Outcome not present at the start^[4]^	Comparability of cohorts on the basis of design or analysis^[5]^	Assessment of outcome^[6]^	Follow-up time^[7]^	Accuracy of follow-up^[8]^	
Singleton et al, 2019^37^	(c)	*	*	*	*	(d)	*	(d)	5 (9)
Silva et al, 2019^36^	(c)	*	*	*	**	(d)	*	*	7 (9)
Korun et al, 2017^22^	(c)	*	*	*	*	(d)	*	*	6 (9)
Schroth et al, 2014^31^	(c)	*	*	*	**	*	(b)	(c)	6 (9)

[1] (a) Truly representative of the average in the community*; (b) somewhat representative of the average in the community*; (c) selected group of users; (d) no description of the derivation of the cohort. [2] (a) Drawn from the same community as the exposed cohort*; (b) drawn from a different source; (c) no description of the derivation of the non-exposed cohort. [3] (a) Reliable record*; (b) structured interview*; (c) written self-report; (d) no description. [4] (a) Yes*; (b) no. [5] (a) Study controls for the most important factor (socioeconomic factors)*; (b) study controls for any additional factor* (season of measurement of 25(OH) D levels, child’s toothbrushing habits, child’s sun exposure, mother’s sun exposure. [6] (a) Independent blind assessment*; (b) record linkage*; (c) self report; (d) no description. [7] (a) Yes (≥12 months)*; (b) no (<12 months). [8] (a) Complete follow-up*; (b) subjects lost to follow-up unlikely to introduce bias (≥80 %)*; (c) follow-up rate <80% and no description of those lost; (d) no statement.

## Discussion

### Summary of Main Findings

Although there are many epidemiological studies dealing with the relationship between low levels of vitamin D and caries prevalence in children, there is still a paucity of evidence from high-quality investigations concerning this subject. Despite the large time span of this review (1998 to 2019), the thirteen studies that fulfilled the inclusion criteria were published in the past eight years (2012 to 2019), and most of them (10 studies) in the past five years (2015 to 2019). Currently, there is a greater emphasis on conducting studies with stricter methodology, which interrelate different variables involved in this association.

The analysis of the included studies showed that several factors of the participants’ geographical and socioeconomic context were considered as predictors of insufficient levels of 25(OH) D and caries. For instance, Chhonkar et al^3^ associated insufficient levels of vitamin D in India mainly with the population's diet (which is poor in calcium), their dark skin, their indoor lifestyle,^12^ and a decreased cutaneous synthesis related to social and religious habits.^7^ Both Chhonkar et al^3^ and Schroth et al^32^ found a statistically significant inverse correlation between vitamin D levels in children and S-ECC. However, Chhonkar et al^3^ did not perform a multivariate analysis. Silva et al,^36^ who investigated fetal and developmental risk factors for caries in an Australian twin study, reported that vitamin D was not a predictor for this dental disease. Nevertheless, the authors verified that non-fluoridated water, maternal obesity, and hypomineralised second primary molars were strongly associated with advanced caries lesions.

Nørrisgaard et al^26^ pointed out that vitamin D3 levels were strongly associated with various socioeconomic and lifestyle factors, and residual confounding factors can never be excluded in observational studies. That RCT reported that vitamin D supplementation during pregnancy was associated with reduced odds of enamel defects.^26^ These results are in agreement with those of Kühnisch et al,^23^ who observed that increased serum 25(OH) D concentrations at 10 years old were significantly associated with lower odds of having enamel defects.

The studies by Herzog et al^13^ and Schroth et al^33^ both included national representative samples, were controlled for confounders, and considered mixed dentition. Nevertheless, while dental examinations were conducted by calibrated dentists in the study by Schroth et al,^33^ in the study by Herzog,^13^ they were conducted by calibrated healthcare professionals who were not dentists; thus, caries experience may have been underestimated in the latter study.^13^ Despite methodological differences, the results of Schroth et al^33^ are consistent with those of Kühnisch et al^23^ and Hujoel.^19^ The meta-analysis of controlled clinical trials by Hujoel suggested that supplemental vitamin D in early life was associated with a 47% to 54% reduced risk of caries.^19^ Although the results of Gyll et al^11^ were not consistent, they supported an inverse relationship between childhood 25(OH) D levels and caries in mixed dentition. Nevertheless, the participants in Gyll’s study came from a population with low-to-moderate caries prevalence and organised oral healthcare, including the participation in mandatory programs for caries prevention.^11^ Regarding the relationship between vitamin D levels in children and their caries experience in permanent dentition, the results were not totally reliable due to a paucity of literature on this topic.

### Limitations

Most of the studies evaluating the association between vitamin D status and caries used a cross-sectional design, which cannot establish a cause and effect relationship, only support an association.

One of the main limitations of this study regards heterogeneity in outcome assessment. Measurement of dental caries experience was not homogeneous across studies, as studies considered prevalence, the dmft/DMFT, dmfs/DMFS indices, or some components of those indices. Moreover, caries was defined differently from study to study, as some authors only considered cavitated lesions, others also included non-cavitated lesions, and others did not report whether some cut-off point was considered for caries definition when the dmft/DMFT index was used. These differences in the assessment of caries experience may compromise the analysis of results.

Likewise, the great diversity in the number of participants and their characteristics, the variability in methods for assessing vitamin D levels and their cut-offs, and the confounding factors considered in each study may limit the comparability of results. Low socioeconomic status is known to be a strong predictor of caries in children.^5,42^ Not only do socioeconomic factors influence oral health, they can also expose children to a higher risk of poor nutrition, thereby possibly impacting their vitamin D levels.^37^ Some studies did not control for certain socioeconomic variables in the regression models, which may have introduced social bias and may be considered a limitation of the results.

Regarding the methodology used for oral examinations in the selected studies, potential bias or imprecision may be detected when the results are compared. Some studies performed only visual examination with a dental mirror,^22,36^ while others used radiographs for caries diagnosis,^11^ tactile examination with the explorer,^3,26^ and/or compressed air before the dental evaluation.^3,11,13,26^ These differences may have led to more precise caries diagnoses in some studies and, consequently, higher caries prevalence and mean number of dmft/DMFT.

Several studies in the literature indicate the importance of vitamin D in caries prevalence, and we verified that vitamin D levels ≥75 nmol/l seem to be more closely related to caries experience than the IOM’s reference values (25(OH) D ≥50 nmol/l), as no consistent results were found with the latter. However, considering the risk of bias of the analysed studies, we could not with certainty determine an effective association between inadequate or insufficient levels of 25(OH) D and higher caries prevalence in children.

Nevertheless, there is a clear need for new scientific studies that allow adopting strict inclusion criteria, creating random sequences, and using a blind methodology regarding participants, examiners, and evaluators, so that a cause-effect relationship can be evaluated.

## Conclusions

There is evidence of an association between low 25(OH) D levels (<75 nmol/l) and caries experience in children. Hence, low vitamin D levels should not be excluded as a potential factor associated with this dental disease. Adequate and sufficient prenatal and childhood levels of 25(OH) D must be ensured in order to promote the child’s oral health. Clinicians should be aware that good prenatal nutrition and early childhood diet influence caries experience in children.
